# A selenosemicarbazone complex with copper efficiently down-regulates the 90-kDa heat shock protein HSP90AA1 and its client proteins in cancer cells

**DOI:** 10.1186/1471-2407-14-629

**Published:** 2014-08-29

**Authors:** Hongtao Shen, Haichuan Zhu, Mowei Song, Yonglu Tian, Yafei Huang, Hui Zheng, Ruiyuan Cao, Jian Lin, Zhenggang Bi, Wu Zhong

**Affiliations:** The First Affiliated Hospital of Harbin Medical University, Harbin, China; Laboratory of Computer-Aided Drug Design & Discovery, Beijing Institute of Pharmacology and Toxicology, Beijing, China; Laboratory Animal Centre, Peking University, Beijing, China; Center for Human Disease Genomics, Peking University, Beijing, China; Synthetic and Functional Biomolecules Center, College of Chemistry and Molecular Engineering, Peking University, Beijing, China

**Keywords:** Selenosemicarbazone, Cell death, Oxidative stress, RNA-seq, HSP90AA1 protein, PIM1, AKT1

## Abstract

**Background:**

The 90-kDa heat shock protein HSP90AA1 is critical for the stability of several proteins that are important for tumor progression and thus, is a promising target for cancer therapy. Selenosemicarbazone metal complexes have been shown to possess anticancer activity through an unknown molecular mechanism.

**Methods:**

The MTT assay, fluorescence-activated cell sorting, and fluorescent microscopy were used to analyze the mechanism of the anti-cancer activity of the selenosemicarbazone metal complexes. Additionally, RNA-seq was applied to identify transcriptional gene changes, and in turn, the signaling pathways involved in the process of 2-24a/Cu-induced cell death. Last, the expression of HSP90AA1, HSPA1A, PIM1, and AKT proteins in 2-24a/Cu-treated cells were investigated by western blot analysis.

**Results:**

A novel selenosemicarbazone copper complex (2-24a/Cu) efficiently induced G2/M arrest and was cytotoxic in cancer cells. 2-24a/Cu significantly induced oxidative stress in cancer cells. Interestingly, although RNA-seq revealed that the transcription of *HSP90AA1* was increased in 2-24a/Cu-treated cells, western blotting showed that the expression of HSP90AA1 protein was significantly decreased in these cells. Furthermore, down-regulation of HSP90AA1 led to the degradation of its client proteins (PIM1 and AKT1), which are also cancer therapy targets.

**Conclusion:**

Our results showed that 2-24a/Cu efficiently generates oxidative stress and down-regulates HSP90AA1 and its client proteins (PIM1, AKT1) in U2os and HeLa cells. These results demonstrate the potential application of this novel copper complex in cancer therapy.

## Background

The 90-kDa heat shock protein HSP90AA1 is a chaperone protein associated with numerous client proteins that are highly expressed in many cancer cells [1, 2]. It stabilizes several cancer-related client proteins including PIM1, AKT, and HIF1A, which are crucial for tumor progression [1]. Thus, HSP90AA1 is an attractive target for cancer therapy [3–5]. Consistently, small molecular inhibitors of HSP90AA1 such as 17-AAG and SNX-2112 show promising results as cancer therapies [6–8]. These compounds bind HSP90AA1 and suppress its chaperone function, leading to degradation of its client proteins.

Copper ion (Cu) is a transition metal that participates in a wide range of cellular processes. As the disruption of copper homeostasis is a pathological feature of cancer cells [9], copper complexes have been investigated for their potential applications as anti-cancer drugs [10]. The anti-tumor mechanisms of copper complexes include cleavage of DNA, generation of oxidative stress, and proteasome inhibition [10]. For instance, the copper complex NSC 689534/Cu exerted its anti-cancer effects by inducing oxidative stress, and counteracting ROS damage by addition of N-acetyl-l-cysteine (NAC) significantly decreased NSC 689534/Cu cytotoxicity. It is not known whether oxidative stress generated by copper complexes can regulate the expression of HSP90AA1 in cancer cells [11]. However, it has been reported that ROS generated by vitamin C and K3 can induce degradation of HSP90AA1, and therefore contributes to inducing cancer cell death [12].

Selenium (Se) is an essential trace element that has been widely studied because of its chemopreventive properties [13]. Selenium compounds can efficiently induce cell death in various cancer cells [14]. For example, metal complexes of selenosemicarbazones (selenium in the place of sulfur) induced apoptosis through the mitochondria pathway in cancer cells [15]. Additionally, nickel (II) complexes of selenosemicarbazones efficiently inhibited metastasis and angiogenesis in breast cancer cells [16].

Herein, we report a novel selenosemicarbazone compound 2-24a that induces cell-cycle arrest in cancer cells. Furthermore, the 2-24a complex with copper (2-24a/Cu) shows significantly increased cytotoxicity compared with 2-24a alone. Detailed analysis showed that 2-24a/Cu induced oxidative stress, accompanied by down-regulation of HSP90AA1 but not HSPA1A. Down-regulation of HSP90AA1 led to degradation of its client proteins, PIM1 and AKT1. These results suggest that 2-24a/Cu could serve as a potential candidate for anticancer therapy.

## Material and methods

### Synthesis of di-2-pyridyl ketone4,4-dimethyl-3-selenosemicarbazide (2-24a)



Di-2-pyridyl ketone 4, 4-dimethyl-3-thiosemicarbazone (4.6 g, 16 mmol) was dissolved in about 30 ml ethanol, and methyl iodide (CH3I) (2.84 g, 20 mmol) was added. The mixture was heated on reflux for 1 h, then mixed with the ethanolic solution of s-methyl-di-2-pyridyl ketone 4,4-dimethyl-3-thiosemicarbazide. A 100-mL, dry three-necked bottle was packed in an ice bath and 1.97 g (25 mmol) Se and 1.13 g (30 mmol) NaBH_4_ were added under a nitrogen atmosphere. These mixtures were heated up before adding 10 mL ethanol, and 1.06 g (10 mmol) NaCO_3_ after 1 h. The ice bath was then removed, and the ethanolic solution of s-methyl-di-2-pyridyl ketone 4, 4-dimethyl-3-thiosemicarbazide was added. The reaction continued for 20 h at room temperature and then 2 mL acetic acid was added. The exhaust was absorbed by an acetic acid lead solution (10%, 400 mL). The reaction solution was filtrated and the solvent was evaporated under reduced pressure. The residue was purified by column chromatography on silica gel using ethyl acetate - petroleum ether (1:2, v/v). The yield was 1.80 g (34%). 1H-NMR (400 MHz, DMSO) δppm: 3.471–3.537 (s,6H) 7.306–7.334 (m,1H) 7.380–7.388 (m,1H) 7.728–7.748 (d,1H) 7.816–7.840 (m,2H) 8.153–8.173 (d, 1H) 8.569–8.579 (d,1H) 8.693–8.705 (d,1H) 15.317 (s,1H) HLPC-MS m/z: 334.3[M + 1] + .IR(cm): 3050w, 2916 m, 1466s, 1430 m, 1325 m, 1305s, 1235s, 1189 m, 1121s, 1052s, 995 m, 892w, 802 s, 739 s, 709 m, 653 m.

### Chemicals and antibodies

Chemicals and antibodies were purchased as follows: H2DCF (Beyotime, S0033), N-acetyl-L-cysteine (NAC, Sigma, A7250), propidium iodide (PI, Sigma, P4170), rabbit anti-HSP90AA1 (Bioworld, BS1181), rabbit anti-HSPA1A (Bioworld, BS6446), rabbit anti-PIM1 (Abcam, ab75776), rabbit anti-AKT1 (Bioworld, BS1978), rabbit anti-GAPDH (Bioworld, AP0063), goat anti-mouse HRP-linked antibody (ZSGB-BIO, ZB-2301), goat anti-rabbit HRP-linked antibody (ZSGB-BIO, ZB-2305), copper (II) chloride (CuCl_2_, Beijing Shiji, China). 2-24a/Cu was freshly prepared by mixing equal molar ratios of 2-24a and CuCl_2_, and diluted to the appropriate concentrations before treatment.

### Cell culture

HeLa, U2os, and other cell lines were obtained from the Cell Bank of the Chinese Academy of Sciences (Shanghai), and were cultured in Dulbecco’s modified Eagle’s medium (DMEM, HyClone, SH30022.01B) supplemented with 10% fetal bovine serum at 37°C in an atmosphere of 5% CO_2_. Cell lines were authenticated based on viability, recovery, growth, morphology, cytogenetic analysis, antigen expression, DNA profile, and isoenzymology by the provider.

### Cell viability

Cell viability was measured by using the MTT assay (Sigma,M5655). About 3000 cells were plated in each well of a 96-well plate at 37°C in a humidified 5% CO_2_ incubator for 12 h before they were treated with the compounds. After treatment, 20 μL MTT (5 mg/mL) solution was added to each well, and the cells were incubated for 4 h at 37°C in a humidified 5% CO_2_ incubator. The medium was removed to stop the reaction and then 150 μL dimethylsulfoxide (DMSO) was added. The plate was measured using a Varioskan Flash reader (Thermofisher, USA) at 490 nm.

### Cell-cycle analysis

About 1.4 × 10^5^ cells were plated in each well of a 6-well plate for 18 h before treatment with Cu, 2-24a, or 2-24a/Cu. After treatment, the cells were collected by trypsinization, fixed in 70% ethanol overnight, washed in PBS three times, re-suspended in 500 mL of PBS containing 0.1 mg/mL Rnase, 50 mg/mL propidium iodide (PI), and 0.2% Triton X-100, and incubated in the dark for 30 min at room temperature. Then, the samples were placed in 12 × 75 Falcon tubes and read on a Becton Dickinson FACStarPLUS. The data were analyzed using Modfit software.

### Analysis of oxidative stress

The intracellular accumulation of oxidative stress was detected using the probe H_2_DCF. Ten micrometers of H_2_DCF was added into the 2-24a-alone, 2-24a/Cu-, or mock-treated cells for 30 min. Cells were then harvested and analyzed by flow cytometry (Becton Dickinson).

### RNA-Seq

Briefly, the cells were treated with 5 μM 2-24a/Cu or 0.1% DMSO for 8 h. Then the total RNA was extracted from both the 2-24a/Cu-treated cells and the control cells by using the RNeasy mini Kit (Qiagen, 74104) according to the manufacturer’s instructions. Total RNA was treated with DNase I (Qiagen, 79254) for 15 min at room temperature to remove residual genomic DNA. The purity and concentration of RNA was assayed by Nanodrop. The quality of RNA was further checked by running a sample of fragmented RNA on a RNA Pico 6000 chip in an Agilent 2100 Bioanalyzer. Total RNA (1 μg) was used to isolate mRNAs with poly(A) tails and then these mRNAs were converted to cDNA using the TruSeq DNA Sample Preparation Kit according to the manufacturer’s protocol. After generation of the target cDNA from U2os cells, sequencing adapters were ligated to short fragments after purifying with a QiaQuick PCR extraction kit, which were then used to distinguish different sequencing samples. Fragments with lengths from 200 to 700 bp were then separated by agarose gel electrophoresis and the fragments were subjected to 15 cycles of PCR amplification. Finally, the prepared libraries were sequenced using Illumina HiSeq™ 2000 before they were checked by q-PCR and analysis in the Agilent 2100 Bioanalyzer. The results obtained from each cell line were matched to the human genome (NCBI Build 36). Results were used for further analysis. Unambiguously mapped results were first used to generate gene counts. Feature counts were normalized using the RPKM (read per kilobases per million aligned reads) method. The RPKM method is able to eliminate the influence of different gene lengths and sequencing discrepancies on the calculation of gene expression. Therefore, the calculated gene expression can be directly used for comparing the difference of gene expression among samples. To detect different expression levels among difference stages, the *P*-value (one-tailed) corresponds to the differential gene expression test (two-sample *t* test with equal variances). Because differentially expressed gene analysis generates large multiplicity problems in which thousands of hypotheses (i.e., whether a particular gene is differentially expressed between the two groups) are tested simultaneously, corrections for false-positive (type I errors) and false-negative (type II) errors are performed using a false discovery rate method.

### Western blot analysis

To analyze protein expression, western blotting was performed as described previously [17].

### Murine sarcoma S180 implanted mice study

Chinese Kun Ming (KM) mice (male and female in equal numbers) of 16–18 g were purchased from the Vital River Laboratories (China) and housed at the laboratory animal center of Peking University (AAALACi-accredited facility). Experiments were undertaken in accordance with the National Institute of Health Guide for Care and Use of Laboratory Animals, with the approval of the Peking University Laboratory Animals Center, Beijing. Murine sarcoma S180 cells were injected subcutaneously into the right oxter region of KM mice (1 × 10^7^ in 200 μL) until the mice adapted to the new environment. After injection, tumors were allowed to develop for 2 days. We then randomly divided the 40 mice into four groups, treated with DMSO in 0.9% saline (control), 1 mg/kg 2-24a, 1 mg/kg CuCl_2_ or with 1 mg/kg of 2-24a/Cu. The mice in the four groups were intraperitoneally injected daily according to their weight. Tumor size was measured using calipers; tumor volume was estimated according to the following formula: tumor volume (mm^3^) = L × W^2^/2, where L is the length and W is the width. Tumor-bearing mice were sacrificed after 10 days. Xenograft tumors were harvested, weighed and then fixed in 4% formalin for histologic study.

### Statistical analysis

Each experiment was repeated at least three times for calculation of standard deviations. The statistical significance of differences was assessed using the Student’s t test in GraphPad prism 5. A P < 0.05 was considered statistically significant.

## Results

### Cytotoxicity of the selenosemicarbazone 2-24a is significantly increased by copper

**Figure 1:**
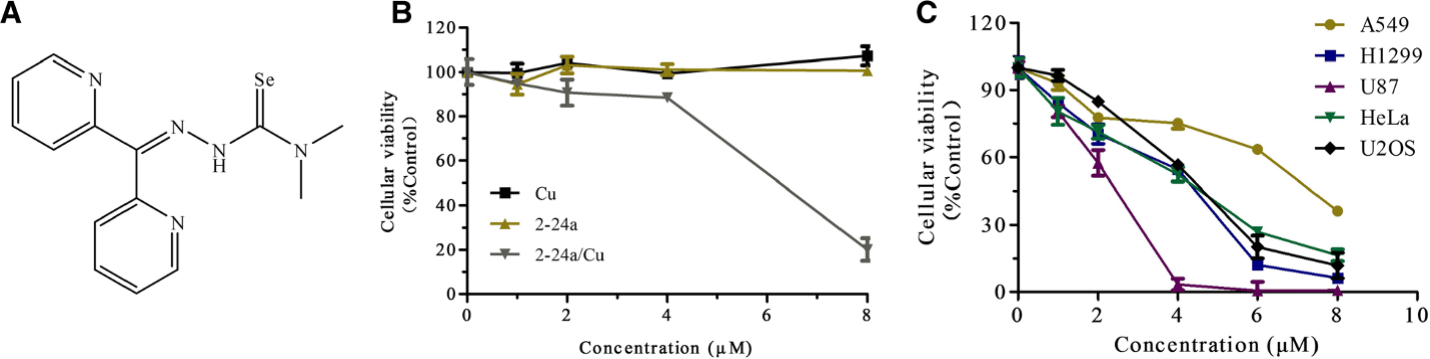
**2-24a/Cu inhibits the viability of cancer cells. (A)** Molecular structure of selenosemicarbazone compound 2-24a. **(B)** Effect of Cu, 2-24a, and 2-24a/Cu on the viability of U2os cells. **(C)** Effects of 2-24a and 2-24a/Cu on the viability of different cancer cells.

**Figure 2:**
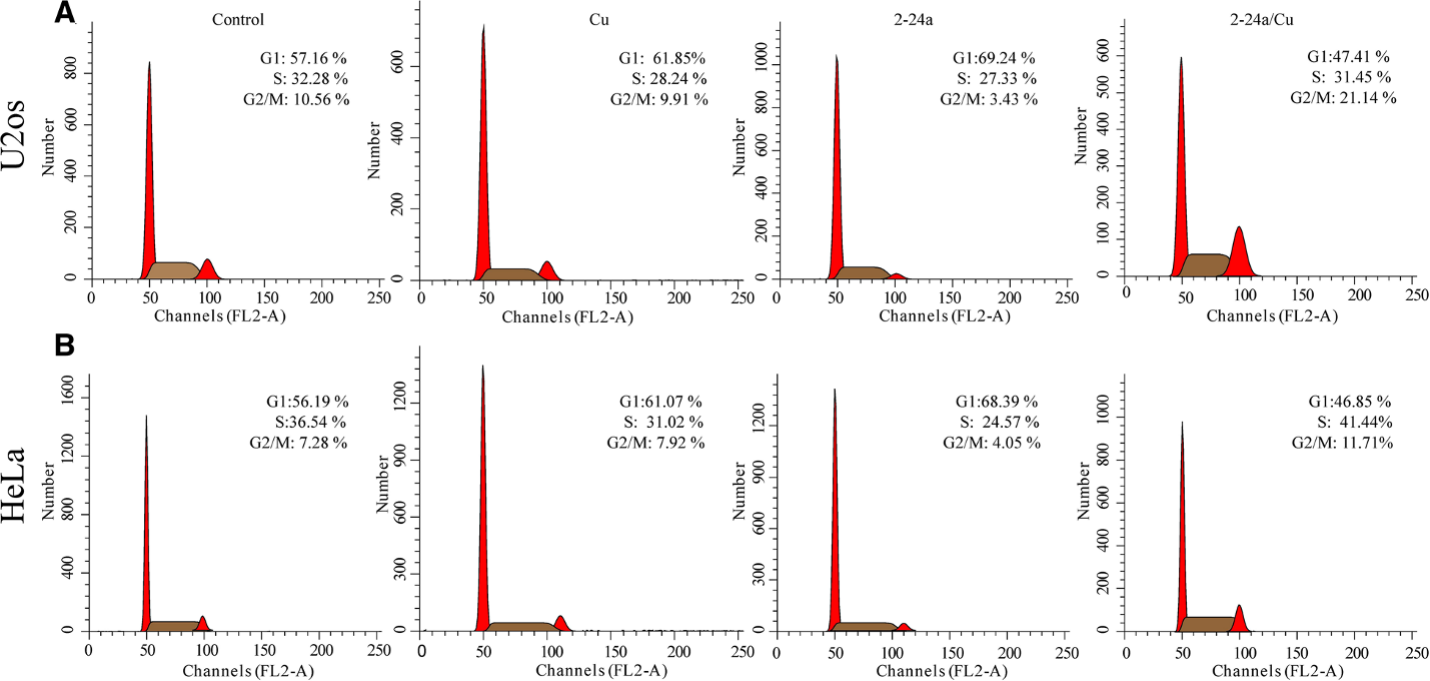
**The effects of copper, 2-24a and 2-24a/Cu on the cell cycles of U2os (A) and HeLa cells (B).**

Compounds belonging to the thiosemicarbazone family have shown anti-tumor potential in different cancer types [18, 19], and complexes with copper had been reported to increase the cytotoxicity of thiosemicarbazones [11, 20]. Their analogs, the selenosemicarbazones, were also reported to have similar effects on cancer cells [15, 16]. A series of novel selenosemicarbazones were synthesized in our laboratory and among these compounds, 2-24a (Figure 1A) complexed with Cu (2-24a/Cu) showed anti-cancer activity in cancer cells. The viability of U2os cells was not significantly decreased by 2-24a (Figure 1B). However, the viability of U2os cells was significantly decreased by 2-24a/Cu in a dose-dependent manner (Figure 1B–C). Similar results were observed in other cancer cell lines (A549 cells, U87 cells, and H1299 cells, Figure 1C). Thus, 2-24a/Cu efficiently reduced cellular viability in various cancer cells.We next investigated the effect of 2-24a/Cu on the cell cycle in cancer cells. 2-24a induced the arrest of the G1 cycle in U2os and HeLa cells, whereas the copper complex 2-24a/Cu induced the increase of the G2/M cycle (Figure 2A–B). These results indicated that 2-24a/Cu had a different effect on the cell cycles compared with 2-24a in cancer cells.

### 2-24a/Cu induces oxidative stress in cancer cells

**Figure 3:**
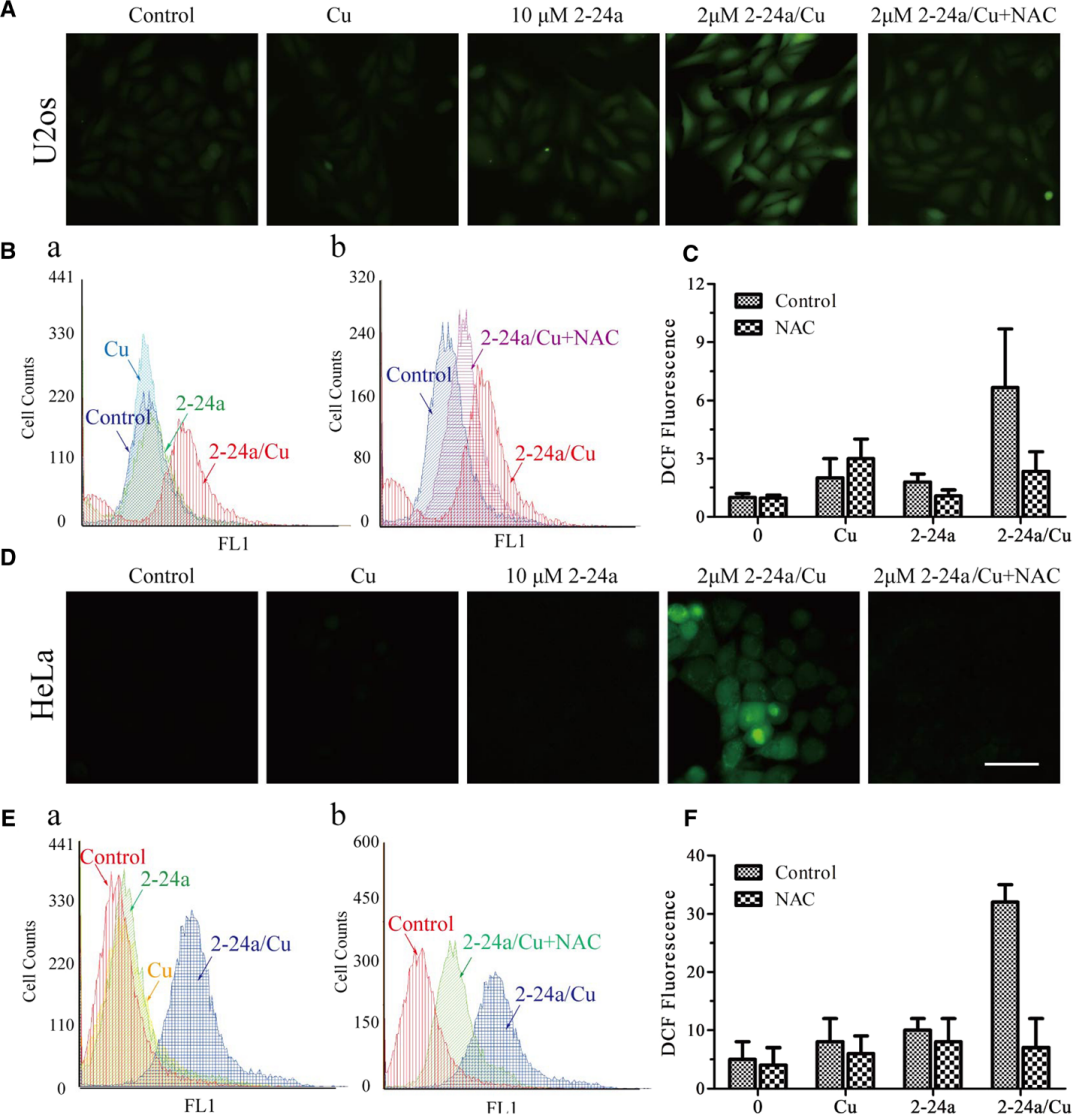
**2-24a/Cu induces oxidative stress in U2os cells and HeLa cells. (A–C)** U2os cells were treated with DMSO, 10 μM copper, 10 μM 2-24a, 2 μM 2-24a/Cu, or an additional 4 mM NAC for 12 h. After incubation with 10 μM H_2_DCFDA, cells were washed and examined by fluorescence microscope **(A)** or FACS **(B)**. The average fluorescent intensity from DCF is indicated **(C)**. **(D**–**F)** HeLa cells were treated as indicated. Means ± SD, n = 3. *P < 0.01.

As copper has been reported to enhance the cytotoxicity of some anti-cancer compounds through induction of oxidative stress [11], we investigated whether 2-24a/Cu acted through a similar mechanism. Conversion of non-fluorescent H_2_DCF to fluorescent DCF was used to assess the intracellular induction of oxidative stress. There was a significant increase of fluorescent DCF in the 2-24a/Cu-treated U2os and HeLa cells, while fluorescent signal changes in cells treated with 2-24a or copper alone were not obvious (Figure 3A–D). We next investigated whether N-acetylcysteine (NAC), a widely used antioxidant, could inhibit 2-24a/Cu-induced oxidative stress. U2os cells or HeLa cells were incubated with 2 μM 2-24a/Cu in the presence of 4 mM NAC. As shown in Figure 3, NAC significantly reduced 2-24a/Cu-induced oxidative stress.

### RNA-Seq analysis of 2-24a/Cu-treated U2os cells

To identify transcriptional changes of genes involved in the process of 2-24a/Cu-induced cell death, 2-24a/Cu- and DMSO-treated cells were subjected to RNA-Seq analysis. Compared with the control cells, 410 genes were up-regulated (fold >1.5-fold, *P* < 0.01), while 603 genes were down-regulated (<-1.5-fold, *P* < 0.01) in the 2-24a/Cu-treated cells. We then analyzed potential signaling pathways in which these genes might be involved. IPA (Ingenuity Systems Inc.) can provide a global functional analysis of RNA-Seq data, which can be used to rank various pathways in the order of statistical significance. Based on our RNA-seq results, the NRF2-mediated oxidative stress response ranks the highest (Table 1). In the NRF2-mediated oxidative stress response pathway, several genes (e.g., *DNAJA4*, *DNAJB*1, DNAJB4, *DNAJB9*, *FOS*, *FOSL1*, *GCLM*, *HMOX1*, *HSPB8, MAFF*, P*IK3R5*, and *SQSTM1*) showed significant transcriptional up-regulation in the 2-24a/Cu-treated cells (fold >2). Additionally, gene transcription in the HIF1α signaling pathway was also significantly up-regulated, including *MAPK15*, *MMP1*, *MMP10*, *MMP25*, P*GF, PIK3R5*, *SLC2A1*, and *SLC2A3*.

**Table 1:** **Top two classes of genes in signal pathways influenced by 2-24a/Cu in U2os cells**

Pathway	Symbol	Gene name	Fold change
NRF2-mediated oxidative stress response	ABCC2	ATP-binding cassette, sub-family C (CFTR/MRP), member 2	1.688
	DNAJA1	DnaJ (Hsp40) homolog, subfamily A, member 1	1.813
	DNAJA4	DnaJ (Hsp40) homolog, subfamily A, member 4	4.526
	DNAJB1	DnaJ (Hsp40) homolog, subfamily B, member 1	4.619
	DNAJB4	DnaJ (Hsp40) homolog, subfamily B, member 4	2.089
	DNAJB6	DnaJ (Hsp40) homolog, subfamily B, member 6	1.865
	DNAJB9	DnaJ (Hsp40) homolog, subfamily B, member 9	2.260
	FOS	FBJ osteosarcoma oncogene	4.602
	FOSL1	FOS-like antigen 1	2.550
	GCLM	Glutamate-cysteine ligase, modifier subunit	2.936
	HERPUD1	Homocysteine-inducible, endoplasmic reticulum stress-inducible, ubiquitin-like domain member 1	1.767
	HMOX1	Heme oxygenase (decycling) 1	6.314
	HSPB8	Heat shock 22 kDa protein 8	2.068
	JUN	Jun proto-oncogene	1.780
	JUNB	Jun-B oncogene	1.982
	MAFF	v-maf musculoaponeurotic fibrosarcoma oncogene homolog F (avian)	3.970
	MAFG	v-maf musculoaponeurotic fibrosarcoma oncogene homolog G (avian)	1.878
	MAP2K6	Mitogen-activated protein kinase kinase 6	-8.079
	PIK3R5	Phosphoinositide-3-kinase, regulatory subunit 5	2.527
	SQSTM1	Sequestosome 1	2.395
	TXNRD1	Thioredoxin reductase 1	1.527
HIF1α Signaling	EGLN3	egl nine homolog 3 (C. elegans)	1.656
	HSP90AA1	Heat shock protein 90 kDa alpha (cytosolic), class A member 1	1.882
	JUN	Jun proto-oncogene	1.780
	MAPK15	Mitogen-activated protein kinase 15	7.962
	MMP1	Matrix metallopeptidase 1 (interstitial collagenase)	3.562
	MMP10	Matrix metallopeptidase 10 (stromelysin 2)	6.231
	MMP25	Matrix metallopeptidase 25	3.564
	PGF	Placental growth factor	3.158
	PIK3R5	Phosphoinositide-3-kinase, regulatory subunit 5	2.527
	SLC2A1	Solute carrier family 2 (facilitated glucose transporter), member 1	2.141
	SLC2A3	Solute carrier family 2 (facilitated glucose transporter), member 3	3.432
	VEGFA	Vascular endothelial growth factor A	1.826
	VEGFC	Vascular endothelial growth factor C	-1.641

We analyzed the genes whose expressions have changed by ±1.5-fold using DAVID software (http://david.abcc.ncifcrf.gov/). The major categories included metal-thiolate cluster genes (including chelation and cadmium genes) and stress-response genes. Metallothionein genes (*MT1B*, *MT1F*, *MT1G*, *MT1H*, *MT1E*, *MT1X*, and *MT2A*) were also significantly up-regulated in the 24a/Cu-treated cells, as were genes encoding HSPA1A and HSP90AA1 proteins (such as *HSP1A*, *HSP1B*, *HSP90AA1*) (Table 2).

**Table 2:** **Category of genes which are significantly up-regulated in 2-24a/Cu-treated cells**

Category	Symbol	Gene name	Fold change
Chelation	MT1B	Metallothionein 1B	7.982
	MT1E	Metallothionein 1E	4.251
	MT1F	Metallothionein 1 F	11.582
	MT1G	Metallothionein 1G	9.692
	MT1H	Metallothionein 1H	14.337
	MT1M	Metallothionein 1 M	10.197
	MT1X	Metallothionein 1X	6.610
	MT2A	Metallothionein 2A	3.335
Stress response	DNAJB1	DnaJ (Hsp40) homolog, subfamily B, member 1	4.619
	DNAJB4	DnaJ (Hsp40) homolog, subfamily B, member 4	2.089
	HSPA1A	Heat shock 70 kDa protein 1A	6.203
	HSPA1B	Heat shock 70 kDa protein 1B	6.351
	HSPA1L	Heat shock 70 kDa protein 1-like	2.607
	HSPA6	Heat shock 70 kDa protein 7 (HSP70B)	10.901
	HSPB8	Heat shock 22 kDa protein 8	2.067
	HSP90AA1	Heat shock protein 90 kDa alpha (cytosolic), class A member 1	1.882
	HSPH1	Heat shock 105 kDa/110 kDa protein 1	3.305
	MAFF	v-maf musculoaponeurotic fibrosarcoma oncogene homolog F (avian)	3.970
	PPP1R15A	Protein phosphatase 1, regulatory (inhibitor) subunit 15A	4.255
	SGK1	Serum/glucocorticoid regulated kinase 1	2.394
	TRIB3	Tribbles homolog 3 (Drosophila)	2.467

### 2-24a-Cu down-regulates HSP90AA1 protein and client proteins (Pim1, Akt1) in U2os and HeLa cells

**Figure 4:**
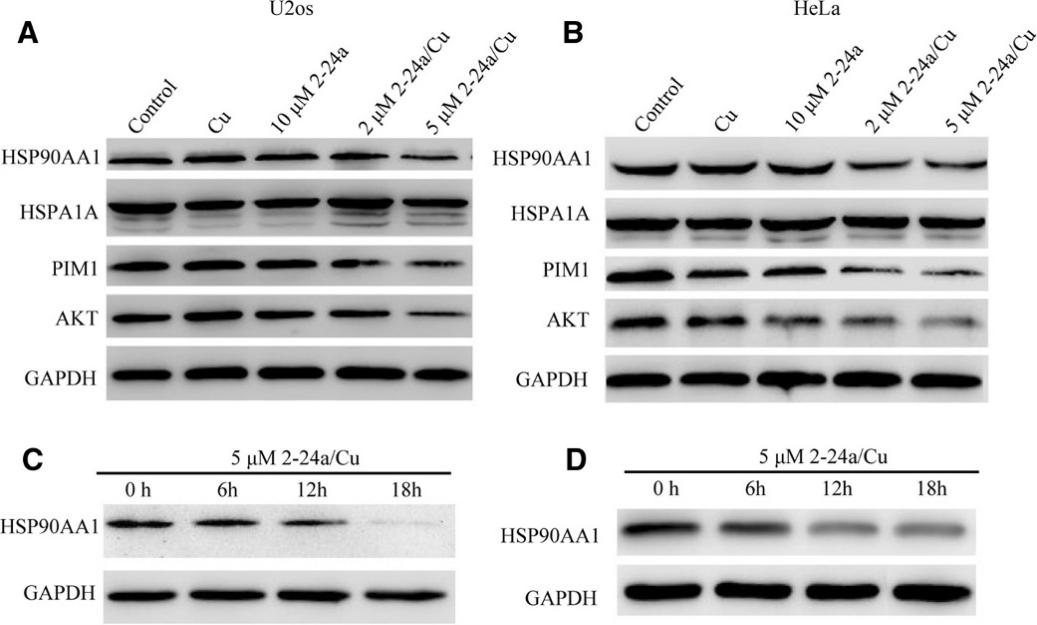
**2-24a/Cu decreases HSP90AA1 protein in U2os cells and HeLa cells. (A, B)** Western blot analysis of HSP90AA1, its client proteins (PIM1, AKT1), and HSPA1A in U2os **(A)** and HeLa **(B)** cells, respectivity. U2os or HeLa were cultured with 0.1% DMSO, 10 μM 2-24a, 2 μM 2-24a/Cu or 5 μM 2-24a/Cu for 8 h. Cells were harvested and lysed for western blotting. **(C, D)** Western blot analysis of HSP90AA1 in U2os **(C)** and HeLa **(D)** cells treated with 5 μM 2-24a/Cu for different time. U2os and HeLa cells were cultured with 5 μM 2-24a/Cu for 0 h, 6 h, 12 h, and 18 h and then harvested and lysed for western blotting.

RNA-seq results revealed a transcriptional increase of heat shock proteins such as HSP90AA1 and HSPA1A. As heat shock family proteins play an important role in the survival of cancer cells, we investigated whether the protein abundance of HSP90AA1 was also increased in the 2-24a/Cu-treated cells. Interestingly, although the transcription of *HSP90AA1* was up-regulated, HSP90AA1 protein was significantly decreased in the 2-24a/Cu-treated U2os and HeLa cells in a dose-and time-dependent manner, while treatment with 2-24a or Cu alone did not show such an effect (Figure 4). The expression of HSP90AA1 in the cells treated with 5 μM 2-24a/Cu decreased to 25% of that in the control cells. Additionally, although the transcription of *HSPA1A* was significantly increased in the 2-24a/Cu-treated cells, the expression of HSPA1A protein was not significantly changed (Figure 4A). These results suggested that the regulation of HSP90AA1 was different to that of HSPA1A in the 2-24a/Cu-treated cells.

As HSP90AA1 protein was significantly decreased in the 2-24a/Cu-treated cancer cells, we next investigated whether the client proteins of HSP90AA1 were also degraded in cancer cells. PIM1 is a client protein of HSP90AA1 in oncogenesis [1], and plays important roles in sarcoma growth and bone invasion [21]. PIM1 protein was significantly decreased in the 2-24a/Cu-treated U2os and HeLa cells (Figure 4A and B). RNA-seq results showed that the transcription of *PIM1* increased by 1.29 fold in the 2-24a/Cu-treated U2os cells, which suggested that the increase of transcriptional *PIM1* would compensate for the decrease of PIM1 protein.

AKT1, another client protein of HSP90AA1 [1], is crucial for survival and proliferation of cancer cells [22]. Cells incubated with 2-24a/Cu also showed the dose-dependent decrease in AKT1 expression (Figure 4A, 4B). Expression of AKT1 obviously decreased in the cells treated with 5 μM 2-24a/Cu compared with the control cells (Figure 4A–B), whereas neither 2-24a nor CuCl_2_ alone treatments decreased AKT1 in HeLa or U2os cells (Figure 4A–B). Additionally, RNA-seq results showed *AKT1* transcription was not changed significantly in the 2-24a/Cu-treated cells. These results indicated that 2-24a/Cu down-regulated HSP90AA1 client proteins (PIM1, AKT1) in U2os and HeLa cells.

### 2-24a/Cu inhibits tumor growth in murine sarcoma S180 implanted mice

**Figure 5:**
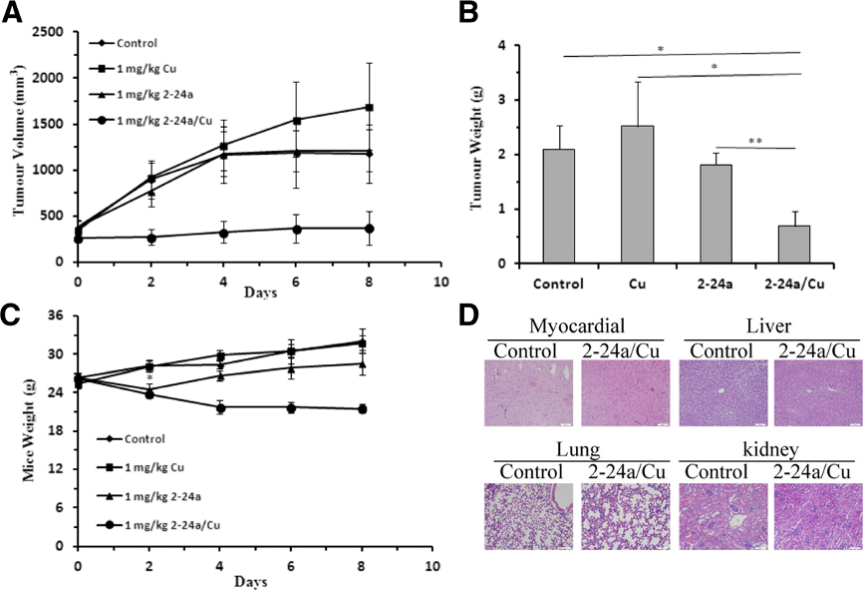
**2-24a/Cu inhibits tumor growth in** ***vivo.*** **(A)** Effects of the control, 2-24a, CuCl_2_ and 2-24a/Cu on tumor volumes in mouse tumor xenografts. **(B)** Effects of the control, 2-24a ,CuCl_2_ and 2-24a/Cu on tumor weight. **(C)** Average weights of mice treated with compounds. **(D)** Representative images of myocardial tissue, liver, lung, and kidney in the control and 2-24a/Cu-treated mice as indicated.

To investigate whether 2-24a/Cu could inhibit tumor growth *in vivo*, murine sarcoma S180 cells [23] were injected (subcutaneously) into the right oxter region of KunMing mice (1 × 10^7^ in 200 μL). Tumors were allowed to develop for 2 days. We then randomly divided the mice into four groups and treated them daily with either vehicle control, 1 mg/kg CuCl_2_, 1 mg/kg 2-24a, or 1 mg/kg of 2-24a/Cu. The tumor sizes (Figure 5A) and the weight of mice (Figure 5C) were measured. At the end of the experiment, the mice were sacrificed and the tumors were removed from the mice and weighed (Figure 5B). 2-24a/Cu significantly inhibited tumor growth *in vivo* by 67% (*P* < 0.01) compared with the controls. Additionally, 2-24a/Cu had little effect on myocardial tissue, liver, lung, and kidney in KunMing mice (Figure 5D).

## Discussion

Compounds from the thiosemicarbazones family have shown promising anti-tumor activity *in vitro* and *in vivo* [24]. Selenosemicarbazones, in which the sulfur atom is substituted by the selenium atom, also showed potential anti-tumor activity [15, 16, 25–28]. Herein, we report that a novel selenosemicarbazone copper complex (2-24a/Cu) can efficiently inhibit cancer cell proliferation and induce cancer cell death, and can serve as the basis for designing other novel anti-cancer selenosemicarbazone compounds.

As expected, a selenosemicarbazone complexed with copper (2-24a/Cu) is significantly more cytotoxic than 2-24a alone in cancer cells because, as reported by other groups, copper can enhance anti-tumor effect of thiosemicarbazones by inducing oxidative stress [20]. Consistently, 2-24a/Cu efficiently generated oxidative stress in cancer cells, and the stress could be efficiently inhibited by NAC. Dp44mT [20], the thiosemicarbazone analog of 2-24a, forms a redox-active copper complex that is responsible for its anti-cancer activity [20]. As copper is elevated in various cancer cells, it is reasonable that 2-24a could be more easily complexed with copper in certain cancer cells than normal cells, leading to cancer cell death. Further research is needed to investigate whether 2-24a could selectively induce cancer cell death via copper-mediated oxidative stress or directly inhibit tumor growth in *vivo*.

We investigated the change of the transcriptome in the 2-24a/Cu-treated U2os cells. RNA-seq results showed that genes that participate in oxidative stress were significantly up-regulated. Additionally, transcription of the genes in the HIF1 signaling pathway were also significantly up-regulated, suggesting the HIF1 signaling pathway could play an important role in regulating copper-mediated cancer cell death.

Metal-thiolate cluster genes and stress-response genes were also up-regulated in the 2-24a/Cu-treated cells, which may play an antagonistic role in the induced cell death. Interestingly, although 2-24a/Cu induced a significant increase in the transcription of Hsp90 family genes (similar to the copper complex of thiosemicarbazone NSC 689534/Cu), HSP90AA1 protein was decreased in the 2-24a/Cu-treated cells. HSP90AA1 is critical for cancer cell metabolism and signal transduction pathways, and inhibition of HSP90AA1 is a promising strategy for cancer therapy [3, 29]. As oxidative stress has been shown to induce HSP90AA1 cleavage in cancer cells [12], it is possible that oxidative stress induced by the copper complex resulted in the degradation of HSP90AA1 in the cancer cells, whereas the transcriptional increase of HSP90AA1 served to compensate for the decrease of HSP90AA1 protein. By decreasing the abundance of HSP90AA1 in cancer cells, 2-24a/Cu could decrease the stability of HSP90AA1 client proteins, many of which are critical in tumor initiation and metastasis. Consistent with this hypothesis, PIM1 (a client protein of HSP90AA1 that affects sarcoma growth and bone invasion [21, 22, 30, 31]) is rapidly decreased in the 2-24a/Cu-treated cells. Similarly, AKT1, which affects cell-cycle arrest and apoptosis [22], is concomitantly decreased in the 2-24a/Cu-treated cells. Because AKT is also important for supporting angiogenesis signaling [32] and 2-24a/Cu has been shown to down-regulate AKT, the latter could prove useful in inhibiting angiogenesis, but the possibility remains to be tested. Expression of other key proteins stabilized by HSP90AA1 such as Bcr-Abl, HER2/Neu (ErbB2), and mutated p53 protein [1], could also be down-regulated by 2-24a/Cu-induced decrease of HSP90AA1, and these collectively contribute to the anti-cancer property of 2-24a/Cu.

## Conclusions

Here, we report that a novel selenosemicarbazone compound (2-24a) and its copper complex (2-24a/Cu) efficiently induced cell-cycle arrest and cell death in cancer cells. 2-24a/Cu was more efficient than 2-24a through generation of copper-mediated oxidative stress. 2-24a/Cu induced the decrease of HSP90AA1 in cancer cells, which is a crucial protein for cancer cell survival. The copper complex (2-24a/Cu) was also an efficient anti-tumor compound in mice. These results suggest that 2-24a/Cu could potentially serve as a basis for a novel cancer therapy.
